# Self‐management recommendations for sickle cell disease: A Ghanaian health professionals' perspective

**DOI:** 10.1002/hsr2.88

**Published:** 2018-09-05

**Authors:** Andrews Druye, Brian Robinson, Katherine Nelson

**Affiliations:** ^1^ School of Nursing and Midwifery University of Cape Coast Cape Coast Ghana; ^2^ Graduate School of Nursing, Midwifery and Health Victoria University of Wellington Wellington New Zealand

**Keywords:** Ghana, health professionals, long‐term conditions, self‐management, sickle cell disease

## Abstract

**Objective:**

To describe self‐management recommendations for sickle cell disease (SCD) care among health professionals who manage SCD in Ghana.

**Method:**

Nine health care professionals (nurses, doctors, and physician assistants) who work in SCD were interviewed. The semistructured interviews were recorded, transcribed, and analysed using the qualitative content analysis method. Self‐management recommendations were conceptualised as preventive health, self‐monitoring, self‐diagnosis, self‐treatment, and self‐evaluation.

**Results:**

Preventive health recommendations were the commonest, where the professionals described similar topics including avoidance of cold temperature, frequent oral hydration, and healthy nutrition. Self‐monitoring recommendations included regular checks for pallor, urine colour, and splenic enlargement. Self‐diagnosis recommendations were captured as warning signs and included pain, fever, unusual feelings, and enlarged spleen. Pain and fever management were the focus of most self‐treatment advice, and there were some self‐treatment recommendations for dactylitis, anaemia, and priapism. There was considerable variation in the strategies recommended for the management of individual SCD‐related problems.

**Conclusion:**

Ghanaian health professionals involved in SCD care provide limited and inconsistent self‐management recommendations. There is a need for the development of SCD standards and guidelines that support effective self‐management. Health professionals working in SCD require continuing education in self‐management.

## 1 INTRODUCTION

Sickle cell disease (SCD) is a major genetic haemoglobinopathy with wide global distribution; sub‐Saharan Africa, including Ghana, is most significantly affected.^1^, ^2^, ^3^, ^4^ This lifelong disorder is characterised by severe pain, chronic anaemia, multiple organ complication, and premature mortality.^5^, ^6^, ^7^, ^8^ Until recently, there has been limited opportunity for cure.^4^, ^9^, ^10^, ^11^ Advances in supportive management including self‐management can improve health status, quality of life, and life expectancy of SCD patients.^12^, ^13^, ^14^, ^15^

Self‐management is integral to managing long‐term conditions (LTCs) such as SCD. It concerns the purposeful performance of specific learned tasks, skills, activities, and behaviours to manage the medical, psychosocial, and life impact of LTCs.^16^, ^17^, ^18^, ^19^ Whilst patients' actions form the foundation of self‐management, health professionals should provide self‐management support, including education and skills, social facilitation, and equipment.^20^, ^21^, ^22^ Effective support by health professionals optimises patients' self‐management, thereby contributing to improvements in patients' health outcomes, the rational use of health resources, and a reduction in health care costs for LTCs such as SCD.^23^, ^24^

Internationally, SCD self‐management research has generally been conducted among patients^25^, ^26^, ^27^, ^28^, ^29^, ^30^, ^31^, ^32^, ^33^, ^34^, ^35^, ^36^, ^37^, ^38^, ^39^, ^40^, ^41^, ^42^ and little has been done to elicit the perspectives of health professionals. Currently, no internationally agreed best practice standards have been published to guide health professionals to support SCD self‐management. However, it is argued that providing advice to patients on certain lifestyle factors, such as avoiding cold weather, rigorous exercise, and dehydration, and engaging with health professionals, is helpful in reducing crisis and complications.^7^, ^12^ In Ghana, as no structured patient‐focused education is provided for SCD self‐management, the education patients receive from health professionals is dependent upon the professionals' knowledge and skills. Until now, no study has examined the self‐management education Ghanaian health professionals provide for SCD care. This paper reports on a study which investigated self‐management recommendations for SCD care among health professionals in Ghana. Understanding self‐management from health professional's perspectives is important to establish what advice is given, whether there is consistency in the messages provided, and to identify areas for further education.

## 2 METHODOLOGY

### 2.1 Design

This study forms the second stage of a 4‐stage sequential mixed method study that investigated self‐management strategies among people living with SCD in Ghana.^43^ The study used a descriptive exploratory approach with interviews and qualitative content analysis.^44^, ^45^ This method was useful, as little is known about SCD self‐management in Ghana and no suitable instrument was located in the literature that could be contextualised to the Ghanaian situation. The study was conducted in a teaching hospital and 3 district hospitals in the Ashanti region of Ghana. The Ashanti region was chosen because until 2016, it remained the only region in Ghana screening newborns for SCD in the public health system and held organised SCD clinics in district hospitals. Ethical approval was obtained from Victoria University of Wellington, the Ghana Health Service, and the Komfo Anokye Teaching Hospital. Self‐management was conceptualised as actions involving preventive health, self‐monitoring, self‐diagnosing, self‐treatment, and self‐evaluation.

### 2.2 Participants

The participants included nurses (n = 6), doctors (n = 2) and a physician assistant. To be eligible for selection, participants had to have at least 3‐month experience in providing clinical care and patient focused education or psychosocial care to patients and families at SCD clinics or the SCD Association (Table 1). The 9 participants were purposively selected. They were the only health professionals available who met the inclusion criteria in the Ashanti region.

**Table 1:** Characteristics of the health professional participants

Name	Years of Experience in SCD	Training in SCD	Role With SCD
NUR1	10 years	Multiple workshops	Education and counselling of patients and parents involved in Newborn Screening for SCD Project (NSSCDP)
NUR2	4 years	Workshop (5 days)	Nurse in‐charge of SCD clinic (District Hospital)
NUR3	19 years	Formal training	Education and counselling of patients and parents, SCD researcher, and member of board of Directors for Sickle Cell Foundation involved in NSSCDP
NUR4	8 years	Some workshops	Education and counselling at SCD clinic/association involved in NSSCDP
NUR5	7 years	Some workshops	Education and counselling at SCD clinic/association involved in NSSCDP
NUR6	5 years	Some workshops	Parental education and counselling for newly diagnosed SCD babies
DR1	10 years	Multiple workshops	Physician in‐charge of SCD clinic (District Hospital) involved in NSSCDP
DR2	10 years	Workshop (5 days)	Former physician in‐charge of SCD clinic (District Hospital)
DR3	4 years	Workshop (5 days)	In‐charge of SCD clinic (District Hospital)

Abbreviations. DR, doctor or physician assistant; NUR, nurse; SCD, sickle cell disease.

### 2.3 Procedure

As phone interviews from New Zealand were the means to conduct the interviews, a local coordinator assisted with recruitment. The coordinator first contacted the potential participants to inform them about the study and distributed consent forms and information sheets. The principal investigator (PI) then followed up with phone calls and e‐mails to solicit agreement to participate in the study.

The interviews were all carried out by the PI and followed a semistructured interview guide. The interview began with an overarching question, “what do you tell your clients with SCD and their families to do for SCD care at home?” Specific questions were asked to capture responses that reflected the topics (preventive health, self‐monitoring, self‐diagnosis, and self‐treatment) in the conceptual framework of the main study. Questions included (1) what they ask patients to do to keep well, (2) how they ask patients to monitor their health, (3) how they advise patients to recognise a sickle cell‐related problem, (4) what actions they advise patients to take when there are problems, and (5) what self‐management practices they have observed in their clinical practice. Each interview continued until information redundancy was observed or when a participant had no more information to share. The interviews were audiotaped and transcribed verbatim.

### 2.4 Analysis

The analysis process involved a 10‐stage iterative process of deductive and inductive approaches informed by 2 publications on qualitative content analysis.^45^, ^46^ The analysis was undertaken by the PI under the supervision of the other 2 authors. The process involved (1) using the conceptual framework (preventive health, self‐monitoring, self‐diagnosis, self‐treatment, and self‐evaluation) to describe self‐management; (2) identifying key concepts/variables as coding categories; (3) operationalising category definitions; (4) selecting units of analysis; (5) becoming familiar with the data—each transcript was read several times to obtain an overall understanding of the recommendations by each participant; (6) using the conceptual framework as a lens, the data were coded into categories using NVivo 10.22; all coding was conducted by the PI (AD) and independently verified by the other authors (KN, BR); (7) selecting text units to corresponding categories; data that did not fit any category were filed under “Other” and reanalysed to identify new subcategories; (8) developing subcategories/new categories; (9) writing the subcategories as text, and reviewing these to test for fitness; and (10) writing findings based on frequency of supporting/nonsupporting evidence of theory/framework.

The deductive approach was warranted, as the study was guided by a conceptual framework and a codebook developed through a review of literature on SCD and other genetic diseases, as well as theories and models of self‐management for chronic diseases and SCD.^43^ Hence, the first 3 steps in the analysis were already established. The inductive approach involved the reading of the text for new categories as well as derivation of themes from the subcategories. The findings are reported using topics and themes derived from the sub‐categories (Appendix A).

## 3 RESULTS

### 3.1 Participants' characteristics

The participants were among the few professionals who have run dedicated SCD clinics in Ghana. Their experience with SCD care averaged 8.6 years (range 4–19) (Table 1). Four nurses had been part of the Newborn Screening for Sickle Cell Disease Project, delivering patient education and counselling. The other 2 nurses and the physician assistant were coordinators for the SCD clinics in their respective district hospitals, and the 2 physicians were coordinators for the SCD clinics in their respective hospitals, delivering clinical care and patient education. In presenting the findings, information that pertain to particular subgroups such as adult or children are specified where the participants indicated these. Where these are not specified, the term patient is used to refer to adults or children with SCD or parents of children with SCD. Quotations are presented by role and number, for example, NUR1. To maintain confidentiality, the physician assistant was coded with a DR number. Tables 1, 2, 3, 4 summarise the categories and themes that were derived from the subcategories and examples of quotations.

### 3.2 Results of self‐management recommendations

All 9 participants reported having discussions with patients about self‐management, but the extent of discussions varied. Preventive health and self‐treatment of painful episodes were the areas that were comprehensively described by the participants.

#### 3.2.1 Preventive health

Preventive health covered health maintenance and preventive care strategies. All 9 participants provided recommendations on aspects of preventive health (Table 2).

**Table 2:** Examples of health professionals' quotes for preventive health

Categories and Themes	Selected Quotes
Healthy nutrition	“And then we also advise them to take well‐nourished diet … They need good nutrition to be able to develop well because their red blood cells are being destroyed at a faster rate almost every 21 days so they need to replenish whatever they are losing.”
Frequent oral hydration	“I think we generally advise them to take a lot a lot of fluids because most of the common complications they come with is VOC.”
Personal hygiene	“We advise them that at home what they should observe is their personal hygiene. You know when they observe personal hygiene, it will help them. So that it will not trigger them.”
Supportive medicines	“When their condition is stable we give them their routine folic acid and penicillin V … we educate them to take it every day. Even when they cannot come to the clinic they should go and buy some and continue taking it.”
Interaction with health professionals	“So, if we take the vaso‐occlusive events it can happen in the form of painful crisis where the patient complains of pains in the joints and … it is very obvious because the person will be screaming and when that happens depending on the level of pain, we advise them to the hospital for hospital management.”
Avoiding extremes of temperature	“we advise them to avoid extremes of temperature, not to take too much cold water, very cold water, not to play in the rain when it's raining.”
Avoiding overactivity	“We also advised them of course to desist from strenuous work, they should know their limit and should know when they have reached their limit”
Avoid diseases that complicates SCD	“and they should also avoid infection, for example malaria and worms so that they wouldn't get anaemia.”
Avoid injuries	“And when they are playing football, the boys they should avoid contact as much as possible...When you playing ball make sure nobody touches your stomach. They should protect themselves.”

Abbreviations: SCD, sickle cell disease; VOC, vaso‐occlusive crises.

##### Health maintenance advice

Health maintenance advice clustered around 2 groups of actions patients were encouraged to perform on a daily basis. The first consisted of actions patients should perform consistently, including maintaining adequate hydration to prevent dehydration, maintaining adequate nutrition, maintaining personal hygiene, and using supportive medication such as folic acid and other prescribed multivitamins. The second set of actions were health professional‐directed actions patients should perform, such as adhering to prescribed medications, attending routine health checks, and seeking clinical care for problems.

All 9 participants reported advising on healthy nutrition. An aspect of the advice concerned the type of diet SCD patients should consume. It was recommended that patients eat a normal diet, which must be “good,” with a selection of a variety of food nutrients, such as carbohydrates, proteins, and fats. A good diet was expressed as “nutritious,” “well balanced,” and “nourishing” and contained green leaves, fruit, and meat. For children, 2 participants recommended breastfeeding and a normal diet and food supplements for those older than 6 months.

The participants spoke of explaining to patients that consuming a good diet would prevent and restore anaemia and undernutrition and improve the immune system. An aspect of the advice given addressed misconceptions about food, especially the consumption of fats and oils. Patients mistakenly ascribed fats and oils as the cause of their jaundice and other problems. The participants also said they caution patients to avoid or reduce the consumption of alcohol and caffeine‐containing food and beverages, and food that elicit allergies, as these can trigger crisis.

All participants, except DR3, said they advised patients on having a liberal oral fluid intake. Water was the most recommended fluid; children are advised to drink other fluids including fruit juices and soft drinks and that fluids be taken in small volumes at frequent intervals. Six participants (DR1, DR3, NUR2, NUR3, NUR5, and NUR6) said they advised patients on the importance of the daily intake of supportive medications, including folic acid, B Complex, multivite, and penicillin prophylaxis. Folic acid supplementation was recommended the most, and many participants commented on its haematogenous abilities. In addition, some participants mentioned advising on the use of penicillin prophylaxis as a means of preventing infectious diseases among SCD children.

Almost all participants considered visits and interactions with health professionals as important for routine health checks, when patients suspect or experience acute problems such as painful crisis or when their home strategies fail to alleviate their symptoms. They highlighted the role of the hospital in helping patients to learn about self‐care from health professionals and from patient role models. The latter were patients who have lived a long life with SCD. The participants noted that poor financial resources served as a barrier to access care. To address financial concerns, NUR2 and NUR4 said they advise patients to register with the National Health Insurance Scheme, and NUR5 reported providing direct financial support by herself.

##### Preventive care advice

Preventive care advice focused on what must be avoided, or performed with caution, to prevent exacerbation of disease. Advice included avoiding extreme temperatures, preventing dehydration, reducing over‐activity, and avoiding food substances that can trigger crisis. Eight participants spoke of advising patients to avoid exposure to cold temperatures, including exposure to cold weather without adequate warm clothing, taking cold baths, or having cold drinks. Many participants spoke of supporting their advice with explanations about the physiological mechanism between cold and painful crisis and emphasised the need for patients to avoid all forms of cold and keep warm. Key among these recommendations was for patients to wear warm clothes most of the time.

Seven participants (DR3 and NUR1–NUR 6) spoke of advising patients to avoid activities involving the use of physical strength or that were vigorous in nature. Strategies to mitigate the impact of over‐activity on the health of SCD patients were noted, including being aware of energy limits, rest breaks, hydration, and exemption from strenuous activities. Three participants reported advising school children to be exempted from strenuous activities at school by providing medical certificates for school authorities. Participants considered it important that patients understood the relationship between over activity, oxygen deprivation, and painful crisis.

The participants also reported advising patients to avoid diseases including malaria and worm infestation, as these can cause crises and anaemia. Many of them spoke about malaria prevention strategies including the use of insecticide‐treated bed nets and chemoprophylaxis. Three participants (DR2, NUR1, and NUR5) advised patients to endeavour to avoid injuries, as wounds on SCD patients are difficult to heal.

#### 3.2.2 Self‐Monitoring

Six participants (DR1, DR2, NUR1–NUR3, and NUR6) outlined physical and physiological indicators that they advise patients to check periodically, including pallor, urine colour, fever, jaundice, splenic enlargement, pain, and general demeanour (for children). The commonest recommendations were checking pallor, urine colour, and fever (Table 3).

**Table 3:** Examples of health professionals' quotes for self‐monitoring and self‐diagnosis

Categories and Themes	Selected Quotes
Self‐monitoring	
Pallour check among children	“they [parents] should be observing the child from time to time for example the conjunctiva and the palm for colour change so that when the baby is getting pale they can compare the baby's palms to theirs they can see the difference in terms of colour.”
Pallour check among adults	“We educate them on how to check the conjunctiva for anaemia that is how they will get to know the warning. They will stand in the mirror and check if your eyes are yellow and you check your conjunctive and it's pale like you look in your palms and you are pale you can report to the hospital.”
Splenic palpation (when there are no enlargements)	“watch out for the spleen. If the child is a new‐born their mothers are taught how to palpate the spleen. If he is a young adult or an adult, again we teach them how to palpate the spleen so that they will be able to determine when their spleen enlarges so they can act appropriately … Then we also teach them how to palpate the spleen of the baby so that they can look out for splenic sequestration which is one of the major complications of SCD.”
Splenic palpation (when there are enlargements)	“those who have splenomegaly, we tell them that they should take note of where the size is, so when they see that it's becoming bigger or its becoming tender they should report to the hospital.”
Growth monitoring	“their [child's] general growth, because, some of them have delayed growth so especially those that are screened, the new‐borns we tell the mothers to be observing their development if by one and half years the child is not walking, they should report to the hospital. Then we will follow her up and see if anything wrong or just the sickle cell.”
Self‐diagnosis	
Fever	“And then if it comes to the infections, of course the infections always exhibit by high fever and once the temperature is beyond 37 degrees [Centigrade] parents should know that their child is running temperature. And we make them aware of the seriousness of temperature and … that there may be an underlying infection such as pneumonia.”
Pallour	“The skill of detecting pallor, they've been taught to look under tongue, look under oral mucosa, look at the conjunctiva, the palm of their children and whenever they see their children are pale, from the usual … steady state like pallor they should bring the child to hospital … the mothers will come, doctor, ‘mehwԑԑ na ni ase no, na ni ase ayԑ fitaa’ [when I look at the eyes they are pale]. We examine and do the HB [haemoglobin], you will be surprised some of them having 4.0, 3.5 and they are walking about. Because of their chronic anaemia … they are able to walk about any how with low HB.”

Abbreviation: SCD, sickle cell disease.

All 6 participants said they advise and show patients how to regularly check for pallor in the conjunctiva, palms, and nail beds. Different techniques were noted for checking pallor among adults and children. The advice on urine checks focused on the type of change to look for and the frequency of checking. Two doctors (DR1 and DR2) specified patients should check their urine daily to see if it is co‐cala colour or dark. Four participants (DR2, NUR2, NUR3, and NUR6) said they advised patients how to check for fever using either a thermometer or the back of the checker's hands. Participants reported advising patients not to consider all fever as malaria and to avoid taking antimalarial medications for all fevers.

Two participants (NUR3 and DR2) reported advising on routine splenic palpation. Different strategies were recommended for patients with apparent splenic enlargement and those without. Three participants (DR2, NUR1, and NUR6) also commented that mothers should be aware that prolonged crying by children could be an indication of painful episodes. NUR6 advised parents to palpate crying children for areas of tenderness. Other recommendations mentioned by different participants included observing for general demeanour, jaundice, and growth of children.

#### 3.2.3 Self‐diagnosis

All the participants used terms such as “emergency signs,” “warning signs,” and “complications” to describe self‐diagnostic indicators. These indicators included fever, changes in urine colour, jaundice, unusual feelings, enlarged or tender spleens, and prolonged crying in children (Table 3).

##### Fever and urine colour changes

There were objective and subjective recommendations to what constitutes the degree of fever that patients should recognise as problematic. Participants either specified a temperature level of 37°C or higher (DR3 and NUR4) or advised patients to note when the body is warm to touch. Change in the colour of urine was the second commonest indicator the participants recommended for self‐diagnosis. Three participants (DR1, DR2, and NUR2) specified dark or “coca cola” urine as an abnormal sign that required attention. The participants related these changes as indicative of physiological abnormalities such as dehydration, vaso‐occlusive crises, and kidney malfunctions and said they urged patients and parents to report immediately to hospital if these should occur.

Four participants (DR2, DR3, NUR2, and NUR5) referred to jaundice as a diagnostic sign and reported advising patients who developed emerging or worsening jaundice to seek immediate care.

#### 3.2.4 Self‐Treatment

Pain and fever management were the focus of most self‐treatment advice, and there were a few recommendations concerning dactylitis, anaemia, pneumonia, and priapism management (Table 4).

**Table 4:** Examples of health professionals' quotes for self‐treatment

Categories and Themes	Selected Quotes
Health professionals recommendations	
Heat application for pain management	“They [children] may not know which parts of the body is paining so they can … keep the baby warm or put some hot compresses around every part of their body or they can even make some warm water and put the baby in the warm water.”
Fever and wound management	“We teach them how to tepid sponge which is one of the first aid in the house is when the child has fever...administration of the drugs, we teach them and they have that skill. Sometimes we teach them a little about … how to treat the wound in the house.”
Priapism management	“So, the male patients we tell them that as soon as they see something like that [priapism] they should drink the fluids and come to the hospital, then we manage.”
Herbal and traditional practices by patients	
Use of herbs	“When they are in pain they boil some herbs and give it to the child … they will give some enema before they come to the hospital especially those who have abdominal distention.”
Weight application for pain	“They will ask that a weight be put on that joint or that part or joint which again, sort of numbs the part of the body and reduces the pain that they are experiencing.”
Scarification to cure disease	“They have also what we call the scarification that are done by traditional medicine men with the belief that sickle cell disease is a *bought disease*, a spiritual disease, and, therefore, by making the scarification on patients and putting in some black quotient can reduce the effects of the disease on the patients through evil manipulation. So they do the scarification and then they sort of give to the spirits.”
Local haematinics for anaemia	“And another person told me something about a drink that is made with ‘Kwawunsusua’ (Turkey Berries). She boils the Kwawunsusua … boil till its green and she drinks it with anything, like she adds sugar, if you want milk you add to make it nice. According to her that is her blood tonic so she takes it like tea every morning.”
Conventional haematinics for anaemia	“We discourage [haematinics] because most blood tonics contain iron and therefore they may be adding more iron to what they already have because we know that when the cells are getting destroyed the iron is stored in their system for use. So if you have not tested to actually show that they are lacking iron, then they are actually adding more iron by taking the blood tonic.”

All participants, except NUR5, reported advising on pain management strategies including the use of analgesics, increased oral fluid intake, heat applications, massage, relaxation, and periodic movement. NUR5 advised that patients experiencing painful events should seek care at the health facility without engaging in any self‐treatment. Among children who cannot verbalise pain, NUR1 recommended applying hot compresses around the whole body of a child if they were crying profusely. Four participants (DR1, NUR3, NUR5, and NUR6) recommended patients to manage fever at home using antipyretic medication and tepid sponging with lukewarm water. Three participants (NUR2, NUR3, and NUR5) mentioned advising parents to treat dactylitis by increasing the child's hydration. They also advised the application of topical analgesic to the affected part, taking oral analgesics, and avoiding vigorous rubbing of the affected area, as this can result in bleeding. DR3 commented that patients experiencing priapism should increase their fluid intake.

The participants also spoke about how they discourage harmful traditional self‐treatment practices that they had observed patients doing. These included advising against the use of raw herbs, binding painful limps, placing heavy objects on painful limbs, and making incisions on painful limbs or distended abdomens. They also caution against the frequent use of haematinics for anaemia and dressing leg ulcers with unsterile equipment.

## 4 DISCUSSION

The 9 health professionals surveyed considered they provide advice on many SCD self‐management topics. However, the content of the advice mainly concerned what people should do to prevent painful crisis rather than remaining well generally, and there was considerable variation in what they reported advising patients to do regarding self‐management. This variation probably occurs because of the lack of international standards and guidelines for the self‐management of SCD.

The professionals' responses mainly focused on preventive health, where many of them described similar topics including temperature, hydration, nutrition, safety with physical activities, taking folic acid supplements, and analgesia. In terms of the specific SCD‐related problems, most of the professionals described similar strategies for pain management, namely, increasing oral fluid intake, applying heat, and topical medicines. As no study has reported self‐management recommendations from the perspectives from health professionals, there are no studies to provide comparison with the current findings. However, the findings for preventive health are similar to research findings on self‐management recommendations and practices from patients and relatives' perspectives in the United States, Brazil, and Jamaica, as well as recommended measures for healthy living and crisis prevention for SCD management.^3^, ^5^, ^7^, ^38^, ^39^, ^40^, ^47^, ^48^

The findings indicate that recommendations for self‐monitoring and diagnosis, using objective methods and measurements such as thermometers, splenic palpation, and self‐assessment tools, are limited in the Ghanaian health professionals' recommendations. In contrast, researchers of SCD self‐management in the United States have focused on objective measurements for pain self‐monitoring to understand pain frequency, characteristics, related symptoms, and home management.^26^, ^28^, ^29^, ^32^, ^33^, ^37^ Typically, these researchers in mostly interventional studies have asked patients to rate their pain using assessment tools, interpret their pain experience, identify related symptoms, categorise their pain and related symptoms to predetermined categories, and record their findings into paper or electronic diaries. Self‐monitoring and diagnoses were facilitated by e‐health technologies in 2 studies.^29^, ^33^ In Jacob et al,^29^ there was live communication between the patient and an advanced practice nurse, specialised in SCD, who remotely monitored patients' entries and contacted patients who required attention. The limited recommendations for self‐monitoring and for objective measurement given indicate that Ghanaian health professionals do not commonly recommend self‐monitoring and diagnostic strategies that involve gathering consistent, measurable evidence of changes in health status. These results may reflect the general lack of formal education regarding managing SCD.

Pain was the only problem addressed by all the participants and was the underlying reason for most recommendations. This finding is consistent with most of the SCD literature, as pain has been the focus of SCD research. Other problems were described mainly in relation to different categories. This variability may reflect the different roles the nurses and doctors have and when they see patients, but also highlights a lack of a systematic approach to self‐management in Ghana. Problems such as undernutrition, anaemia, malaria, and pneumonia were described in relation to preventive health, whereas abdominal pain, vomiting, splenic enlargement, impaired growth, changes in urine colour, and jaundice were described in relation to diagnosis. Complications described in terms of self‐treatment were pain, fever, dactylitis, and priapism. Apart from pain, where similar strategies were described by most participants, less than half of the participants described recommendations for all the other complications, with wide variations in the types of strategies recommended. Given that pain was the only complication described by all professionals, patients in Ghana could be missing out on important advice concerning other SCD‐related conditions.

There are 4 main limitations to this study. Firstly, a semistructured interview approach was used, asking participants to share what they did. Using a survey approach with closed questions may have revealed different answers about practices. Secondly, some of the professionals' roles in SCD care have been reduced with the closure of the screening programme in 2010. Hence, their ability to recall what they recommend to patients and parents may be limited. Thirdly, limited distinction was made by the professionals regarding recommendations for adults and children in this study. We acknowledge that some recommendations may be more applicable to one group than the other. Future research is required to categorise recommendations for different age groups. Finally, saturation of findings was not achieved. However, the study did recruit all health professionals in the Ashanti region who met the inclusion criteria. Expanding the study to other regions might have produced additional information and generated less variability amongst the advice given.

## 5 DISCUSSION

The lack of international standards and guidelines for self‐management of SCD results in arbitrary self‐management advice being given by Ghanaian health professionals to patients with SCD. As self‐management actions by patients can improve general health and well‐being as well as minimise the likelihood of people with SCD developing crises, there needs to be a more consistent approach to advising on self‐management actions and strategies.

## 6 PRACTICE IMPLICATIONS

Given the vast experiences of the professionals in SCD care, the professionals' recommendations in this study provide insight into important topics and measures that can be helpful in improving patients' self‐management and health outcomes. The recommendations provided are likely to be useful for patients and health professionals who are not directly involved in SCD care to improve their knowledge and care for SCD. Guidelines for structured self‐management support are required to assist health professionals to effectively support patients' self‐management in Ghana and other countries with similar health systems. The topics identified in this study can be considered in developing guidelines. More research into health professionals' knowledge and patients' preferences for SCD self‐management is also required internationally to understand what is important to teach patients.

## FUNDING

This study was financed by the Victoria University of Wellington.

## CONFLICT OF INTEREST

None.

## AUTHOR CONTRIBUTIONS

Conceptualisation: Andrews Adjei Druye, Katherine Nelson

Formal Analysis: Andrews Adjei Druye, Katherine Nelson, Brian Robinson

Funding acquisition: Andrews Adjei Druye, Katherine Nelson

Methodology: Andrews Adjei Druye, Katherine Nelson

Investigation: Andrews Adjei Druye

Project Administration: Andrews Adjei Druye, Katherine Nelson

Supervision: Andrews Adjei Druye, Katherine Nelson

Validation: Andrews Adjei Druye, Katherine Nelson, Brian Robinson

Visualisation: Andrews Adjei Druye, Katherine Nelson, Brian Robinson

Writing – Original Draft Preparation: Andrews Adjei Druye, Katherine Nelson, Brian Robinson

## APPENDIX A

### A.1 List of categories and codes

**Table:**

Categories	Codes
Preventive health recommendations	Folic acid supplementation
	Vitamin supplements. eg, B'CO and Multivite
	Haematinics (for preventive treatment)
	Malaria prophylaxis
	De‐wormer
	Frequent hydration
	Healthy Nutrition
	Avoid strenuous activities
	Dress to stay warm
	Avoid cold (baths, baths, environment)
	Use of mosquito net
	Uses of mosquito spray
	Attend clinical appointments
	Penicillin prophylaxis
Self‐monitoring recommendations	Check urine colour
	Check for jaundice
	Look for paleness
	Check temperature
	Palpate the abdomen
	Look for signs of diarrhoea and vomiting
	Listens to body
	Keeps a diary or records
	Check urine colour
Self‐diagnosis recommendations	Painful episodes
	Urine colour changes
	Jaundice
	Fever
	Dizziness/ collapse
	Diarrhoea
	Vomiting
	Pallour
	Swollen joints
	Abdominal pains
	Difficulty in breathing
	Painful erection of the penis (males)
Self‐treatment recommendations	Analgesics
	Increased fluid intake
	Heat application to affected part
	Applying cold to affected part
	Massaging
	Rest
Health professional observations of patient's self‐treatment	Tied affected limb
	Applied weight to affected limb
	Use of traditional medicines
	Reduced activities
Items recommended for patients to keep at home	
	Analgesics (oral/topical)
	Folic acid
	Thermometer
	Warm clothing
	Wound dressing equipment
	Health education materials
